# A qualitative examination of the content validity of the EQ-5D-5L in patients with type 2 diabetes

**DOI:** 10.1186/s12955-015-0373-7

**Published:** 2015-12-01

**Authors:** Louis S. Matza, Kristina S. Boye, Katie D. Stewart, Bradley H. Curtis, Matthew Reaney, Amanda S. Landrian

**Affiliations:** Outcomes Research, Evidera, 7101 Wisconsin Avenue, Suite 1400, Bethesda, MD 20814 USA; Global Patient Outcomes and Real World Evidence, Eli Lilly & Co., Indianapolis, IN USA; Global Medical Affairs, Eli Lilly & Co., Indianapolis, IN USA; ERT, Peterborough, UK

**Keywords:** EQ-5D, Type 2 diabetes, Content validity, Qualitative research, Cognitive interviews

## Abstract

**Background:**

The EQ-5D is frequently used to derive utilities for patients with type 2 diabetes (T2D). Despite widely available quantitative psychometric data on the EQ-5D, little is known about content validity in this population. Thus, the purpose of this qualitative study was to examine content validity of the EQ-5D in patients with T2D.

**Methods:**

Patients with T2D in the UK completed concept elicitation interviews, followed by administration of the EQ-5D-5L and cognitive interviewing focused on the instrument’s relevance, clarity, and comprehensiveness.

**Results:**

A total of 25 participants completed interviews (52.0 % male; mean age = 53.5 years). Approximately half (52 %) reported that the EQ-5D-5L was relevant to their experience with T2D. When asked if each individual item was relevant to their experience with T2D, responses varied widely (24.0 % said the self-care item was relevant; 68.0 % said the anxiety/depression item was relevant). Participants frequently said items were not relevant to themselves, but could be relevant to patients with more severe diabetes. Most participants (92.0 %) reported that T2D and/or its treatment/monitoring requirements had an impact on their quality of life that was not captured by the EQ-5D-5L. Common missing concepts included food awareness/restriction (*n* = 13, 52.0 %); activities (*n* = 11, 44.0 %); emotional functioning other than depression/anxiety (*n* = 8, 32.0 %); and social/relationship functioning (*n* = 8, 32.0 %).

**Conclusions:**

The results highlight strengths and potential limitations of the EQ-5D-5L, including missing content that could be important for some patients with T2D. Suggestions for addressing limitations are provided.

## Background

Generic preference-based instruments are frequently administered to estimate utility values for type 2 diabetes (T2D) health states for use in cost-utility modeling. The most commonly used of these instruments in the European Union is the EQ-5D, which has been used to estimate utilities of patients with T2D in large surveys across multiple countries [1–6]. A recent systematic review found over 50 published articles reporting EQ-5D values for patients with T2D from 1987 to 2009, including longitudinal studies, large cross-sectional surveys, and randomized clinical trials [7].

It is likely that the EQ-5D is commonly used partly due to the NICE Guide to the Methods of Technology Appraisal, which specifies a preference for utilities based on the EQ-5D in order to maximize “consistency across appraisals” [8]. However, the NICE guide adds that utilities derived via other methods may be acceptable for use in cost utility modeling when EQ-5D utilities are not “available” or “appropriate.” The recently updated version of the guide suggests that qualitative data may be useful for examining the appropriateness of the EQ-5D. To support a claim that the instrument is inappropriate, NICE suggests “qualitative empirical evidence on the lack of content validity for the EQ-5D should be provided, demonstrating that key dimensions of health are missing. This should be supported by evidence that shows that EQ-5D performs poorly on tests of construct validity and responsiveness in a particular patient population.” Thus, while quantitative psychometric data is a key component of instrument evaluation, this quote indicates that NICE also considers qualitative evidence on content validity in the target population to be important when evaluating appropriateness of the EQ-5D.

Although content validity of utility assessment tools is not frequently examined, it is considered a critical measurement property to be documented as part of the development and validation of patient-reported outcome (PRO) measures [9–11]. Content validity is the extent to which an instrument assesses the relevant and important aspects of the concept it was designed to measure [12], and it is generally established through two steps of qualitative research with patients [9, 13, 14]. In the concept elicitation step, patients help identify concepts and wording that will be used to shape the items of a PRO measure [10]. After drafting the instrument, the second step is to conduct cognitive interviews in which patients complete the instrument and comment on its relevance, clarity, and comprehensiveness [11]. This qualitative research should be conducted in samples of patients from the target population to establish content validity for a particular use because an instrument may have content validity in one disease, but omit concepts that are important for assessment of another disease.

Psychometric properties of the EQ-5D-3L have been examined in a wide range of quantitative studies, and the instrument is generally found to have acceptable reliability, convergent/divergent validity, and known-groups validity [7, 15–18]. Responsiveness to change is also frequently demonstrated, although responsiveness does not appear to be entirely consistent across studies [7, 17, 18]. Despite the strengths, it is common for authors to report ceiling effects [7, 17, 18], which could limit accuracy of assessment of patients with milder T2D. In sum, quantitative data generally support the EQ-5D, while noting some potential limitations in patients with T2D.

Despite the widely available psychometric data on the EQ-5D, little is known about the content validity of the instrument in patients with T2D. The field of PRO instrument development has reached a consensus that content validity cannot be established via quantitative psychometric analyses, as highlighted in the FDA PRO guidance [9]. For example, it is possible for an instrument to demonstrate good reliability and construct validity in a quantitative analysis without being sufficiently comprehensive or relevant for a specific target population. Qualitative research has not previously been conducted to examine content validity of either the EQ-5D-3L or the more recent EQ-5D-5L in patients with T2D. Thus, the purpose of the current qualitative study was to examine the content validity of the EQ-5D-5L in patients with T2D.

## Methods

### Overview of study design

This qualitative study involved semi-structured, one-on-one, in-person interviews with individuals diagnosed with T2D. Interviews began with concept elicitation, designed to identify the impact of T2D on quality of life. Then, participants were asked to complete the EQ-5D-5L, and a cognitive interview was conducted, focusing primarily on the relevance and comprehensiveness of the instrument for patients with T2D.

### Study participants

Participants were recruited via advertisements in two newspapers and the website Gumtree.com. To be eligible for the study, participants were required to be (1) residing in the UK; (2) at least 18 years old; (3) diagnosed with T2D by a medical professional; and (4) able to provide proof of T2D; and (5) able to understand interview procedures.

A total of 55 individuals were reached for screening by telephone. Of the 55 screened participants, 45 were eligible, including 29 who were scheduled for interviews and 16 who were not scheduled. Of these 16, three were not scheduled because they were unavailable on the dates when interviews occurred. The other 13 were not scheduled because they were in a group whose recruitment target had already been met at the time of their screening. The aim of these targets was to ensure that no demographic group was over-represented (e.g., age and gender groups), while recruiting a sample with representation from three treatment groups (no medication treatment, oral medication only, injectable medication). Of the 29 individuals who were scheduled, 26 attended interviews. One participant was unable to provide direct answers to the interview and therefore could not complete the study procedures. Thus, a total of 25 valid interviews were completed.

### Qualitative interview procedures

Interviews were conducted in October 2013 in London, UK. Participants provided confirmation of T2D diagnosis by presenting diabetes medication with their name on the packaging (*n* = 20) or demonstrating knowledge of the symptoms and tests that led to their diagnosis (*n* = 5). Procedures and materials were approved by an independent Institutional Review Board (Ethical & Independent Review Services - 13118-01), and participants provided written informed consent prior to completing study procedures.

Interviews were conducted according to a semi-structured interview guide, which was designed to evaluate the content validity of the EQ-5D-5L in patients with T2D. First, participants were asked about their T2D diagnosis and treatment. The second phase of the interview was concept elicitation, in which participants were asked about the impact of T2D on quality of life (e.g., How does T2D or its treatment impact your quality of life?). This open-ended section was designed to elicit concepts that arose spontaneously from the respondents, without suggestions from the interviewer regarding possible domains of impact. Third, participants were asked to complete the EQ-5D-5L.

After completing the questionnaire, cognitive interview procedures were followed to assess understanding of the instrument’s instructions, items, and response options, as well as the relevance and comprehensiveness of the content for patients with T2D. Participants were asked how they understood the instructions, how they interpreted each item, and how they selected a response. The order of items discussed in each interview was varied to avoid potential bias of order effects. Participants were also asked whether the questionnaire was missing potentially important content (e.g., Was anything important about the impact of T2D missing from this questionnaire? Does T2D impact your quality of life in any other ways that are not captured by this questionnaire?).

### Measures

#### EQ-5D-5L

The EQ-5D questionnaire is a generic, preference-based health-related quality of life (HRQL) instrument designed to yield health state utilities that may be used in cost-utility modeling [19]. The original EQ-5D-3L contains five items to assess functioning in the following domains: mobility, self-care, usual activities, pain/discomfort, and anxiety/depression. In response to concerns regarding the original EQ-5D-3L, the EQ-5D-5L questionnaire was developed to improve the instrument’s sensitivity and reduce ceiling effects [20]. The EQ-5D-5L assesses the same five domains as the original version, but with slightly revised phrasing and five response options per item instead of three. The five response options are: no problems, slight problems, moderate problems, severe problems, and extreme problems. There is also a visual analog scale (VAS), which asks participants to rate their current health from 0 (the worst health you can imagine) to 100 (the best health you can imagine).

#### Sociodemographic and clinical form

A demographic and clinical information form was completed by participants prior to the interview. This form included questions on age, sex, ethnicity, living situation, employment, education, diabetes-related health, and general health.

### Qualitative data analysis

All interviews were audio recorded and transcribed so that the transcripts could be analyzed using ATLAS.ti, software designed for analysis of qualitative data. A coding dictionary was developed based on the themes and concepts that emerged during the interview discussions. Two staff members independently coded the first cognitive interview transcript and codes were reconciled to examine agreement and disagreement between coders. After it was established that coders agreed on the meaning and application of the codes, subsequent transcripts were each coded by one of these two coders.

During the qualitative analysis, the codes specified in the dictionary were assigned to relevant text in each transcript, thus identifying quotes within each concept or theme. Throughout this process, coders suggested additional codes based on content of the transcripts, and new codes were added to capture these emerging concepts. This coding process resulted in sets of quotes categorized by thematic code, and saturation was documented. Saturation is defined as a point at which no substantially new themes, concepts, or terms are introduced as additional interviews are conducted [14].

## Results

### Sample description

The sample of 25 participants was 52 % male (*n* = 13), with a mean age of 53.5 years (Table 1). The majority of participants reported ethnic/racial background as white (60.0 %), and more participants reported being married (56.0 %) than single (28.0 %) or divorced (8.0 %). Most participants reported being employed (44.0 % full time and 12.0 % part time). Less than half of the sample had completed a university degree (36.0 %). When asked to report health conditions other than diabetes, the most common responses were depression (*n* = 7, 28.0 %), hypertension (*n* = 6, 24.0 %), arthritis (*n* = 2, 8.0 %), and stroke (*n* = 2, 8.0 %).

**Table 1:** Demographic and clinical characteristics

Characteristics	Participants (*n* = 25)
Age (mean, SD)	53.5 (8.9)
Gender (n, %)	
Male	13 (52.0 %)
Female	12 (48.0 %)
Racial/Ethnic Background (n, %)	
White	15 (60.0 %)
Black/African/Caribbean	4 (16.0 %)
Asian	3 (12.0 %)
Mixed/Multiple Ethnic Groups	3 (12.0 %)
Marital Status (n, %)	
Single	7 (28.0 %)
Married	14 (56.0 %)
Divorced	2 (8.0 %)
Other^b^	2 (8.0 %)
Employment Status^a^ (n, %)	
Full-time work	11 (44.0 %)
Part-time work	3 (12.0 %)
Disabled	4 (16.0 %)
Other^c^	8 (32.0 %)
Education Level (n, %)	
University/degree (BA, BSc) or postgraduate degree (MA, PhD, PGCE)	9 (36.0 %)
No university degree	16 (64.0 %)
Current Treatment (n, %)	
Oral only	12 (48.0 %)
Oral and injection	8 (32.0 %)
Diet and exercise	5 (20.0 %)

^a^Not mutually exclusive

^b^Marital status: Other = One participant indicated their marital status as “Live with partner,” and one reported being widowed

^c^Employment status: Other = Two participants reported employment status as “student,” two participants were “unemployed,” two participants were “retired,” one participant was “self-employed,” and one participant was a “homemaker/housewife”

Current treatment regimens for T2D included oral medication (*n* = 12; 48 %), oral plus injectable medication (*n* = 8; 32 %), and diet/exercise (*n* = 5; 20 %). The 20 participants on oral and/or injectable treatment regimens brought proof of medication to the interviews. The five participants who were not taking medication were asked to describe their symptom history, diagnosis process, and disease management strategies. All five of these participants provided information at a level of detail strongly suggesting that they were honestly reporting their diagnoses. More than half of the participants (*n* = 14, 56.0 %) reported that they had experienced daytime hypoglycemia, and some reported experiencing nocturnal hypoglycemia (*n* = 7, 28.0 %). The majority of participants reported that they checked their own blood glucose levels at least once per week (*n* = 16; 64 %).

No new concepts relevant to the current study goals were raised in the final interview. Thus, it appeared that saturation was reached, and the current sample size was considered sufficient for this qualitative study.

### Concept elicitation: impact of type 2 diabetes on quality of life

Concept elicitation, in which participants were asked about the impact of T2D on their quality of life, was conducted prior to administering the EQ-5D-5L. Participants spontaneously reported a variety of impacts including the examples in Table 2.

**Table 2:** Type 2 diabetes impact commonly reported by participants

Domain of impact	Number of participants reporting each impact	Example quotations
Dietary restrictions	17	• You miss being able to just eat and drink what you want without thinking about it, because you know it’s always in your mind.
Limitations on their activities	9	• I used to go swimming…I don’t do the sports anymore.
		• I’m a bit slower in daily activities than I used to be.
Decreased energy	7	• By the afternoon I am tired… I can’t keep a conversation up because I just want to go to sleep.
Emotional	7	• Because of my illness, I get grumpy… I’m less tolerant than I used to be.
Social	8	• …makes me very more withdrawn.
		• I used to go out and meet up with friends and have no cares or worries, but now I’m concerned that I have to be back, take my medication.
Relationship	3	• It’s detracted pleasure from, um, when I go out with my husband.

### Cognitive interviews (including relevance of the EQ-5D-5L to type 2 diabetes)

Following concept elicitation, participants completed the EQ-5D-5L and were asked about the questionnaire. When asked to rephrase the instructions, all participants demonstrated a good general understanding, although six participants failed to notice the recall period of “today” and responded based on a different recall period. Only one of the 25 participants reported difficulty with the response options, reporting difficulty distinguishing between slight and moderate problems.

Approximately half (52.0 %) of the sample reported that the EQ-5D-5L was relevant to their experience with T2D (Table 3). One participant reported that the EQ-5D-5L was not relevant to T2D (*“I mean I don’t think that really applies – does it, if you’ve got diabetes.”*). Other participants indicated that the questionnaire was not relevant to their experience with T2D, but may be relevant to other patients (*n* = 1; e.g., *“people with T2D do have those problems… but for me at the moment, no, I'm not experiencing those problems.”*), may be relevant for severe diabetes patients (*n* = 3; e.g., *“I know people who’ve had diabetes that are much more extreme…a lot to the things you’ve asked here they’ve suffered from… I’ve got very little impact on these.”*), or was relevant to general health but not diabetes (*n* = 2; e.g., *“Relevant to my experience in how I am now in my life at my age and my physical health…not specifically relevant or irrelevant to T2D.”*).

**Table 3:** Item relevance (*N* = 25)

		Individual items^b^				
	Overall EQ-5D^a^	Mobility	Self-care	Usual activities	Pain/Discomfort	Anxiety/Depression
Relevant	13 (52.0 %)	9 (36.0 %)	6 (24.0 %)	12 (48.0 %)	9 (36.0 %)	17 (68.0 %)
Not Relevant	1 (4.0 %)	1 (4.0 %)	2 (8.0 %)	1 (4.0 %)	3 (12.0 %)	0 (0.0 %)
Mixed Responses	10 (40.0 %)	12 (48.0 %)	11 (44.0 %)	6 (24.0 %)	10 (40.0 %)	3 (12.0 %)
Relevant for others, but not for me	1	2	4	1	3	2
Relevant for more severe patients	3	6	5	4	5	0
Other mixed responses (e.g., yes and no, potentially, some of them)	4	1	2	0	2	0
Relevant to general health, but not diabetes specific	2	3	0	1	0	1
Unclear Answer^c^	1 (4.0 %)	3 (12.0 %)	6 (24.0 %)	5 (20.0 %)	3 (12.0 %)	5 (20.0 %)
Question Was Not Asked^d^	-	-	-	1 (4.0 %)	-	-

^a^“Were these questions relevant to your experience as a person with type 2 diabetes?”

^b^“Do you think this question is relevant to type 2 diabetes and its impact on most patients?”

^c^Answers were considered “unclear” if the participant did not answer the question directly or provide clear indication of whether the item is relevant to type 2 diabetes

^d^One participant was not asked about the Usual Activities item

Participants were also asked about the relevance of each individual EQ-5D-5L item for patients with T2D. Responses are summarized in Table 3 and by item below:

#### Mobility

Twenty-two of the 25 participants provided clear opinions on the relevance of mobility to T2D, while the other three participants did not clearly indicate whether the item was relevant. Nine participants indicated that the mobility item was relevant to T2D. One participant said that this item was not relevant to “*a normal diabetic patient*.” Nearly half of the participants provided qualified or mixed answers regarding relevance of the mobility item. For example they said it may be relevant for other patients (*n* = 2; e.g., *“probably to other people, but not to me.”*), patients with severe diabetes (*n* = 6; e.g., *“Well if it’s severe, diabetes also after a long, long time.”*), or during a hypoglycemic episode (*n* = 1; e.g., *“Well, it depends on your sugar levels, because I know it’s hard for me to see when I get hypoglycemic… then I’m not stable.”*). Three respondents said the item is relevant for general health, but is not specific to diabetes (e.g., *“I think that question is relevant to any illness.”*).

#### Self-care

Nineteen of the 25 participants provided clear opinions on the relevance of self-care to T2D, while six did not clearly indicate whether the item was relevant. Six participants said the self-care item was relevant to T2D (e.g., *“if you’re really, really tired, you can’t stand up…it can take over you and then you just wouldn’t have no energy to even get up and wash or dress.”*). Two participants (8.0 %) said that self-care was not relevant to T2D (e.g., *“it’s got nothing to do with diabetes”*). Other participants (*n* = 11) provided qualified responses indicating that the self-care item may be relevant for other diabetes patients (*n* = 4; e.g., *“I’ve no problems. But I do know diabetics who have problems with self-care.”*), patients with severe diabetes (*n* = 5; e.g., *“Maybe in ones who have very advanced, long-term diabetes.”*), or relevant to a degree (*n* = 2; e.g., *“There's some degree of relevance.”*).

#### Usual activities

Nineteen participants provided clear opinions on the relevance of usual activities to T2D, five did not clearly indicate whether the item was relevant, and one participant was not asked about the relevance of this item. Usual activities received the second highest number of endorsements as relevant to T2D (*n* = 12). Only one participant said that this item was not relevant to T2D. Six participants provided mixed responses about the relevance of usual activities. For example, one said that depression may interfere with usual activities for some patients with T2D. Four participants indicated that the usual activities item may be relevant to severe T2D (e.g., *“depending on the severity of the diabetes, I can imagine it would have an effect on work;” “I suppose if my diabetes really progressed and it affects my eyesight that would be different”*).

#### Pain/discomfort

Twenty-two participants provided clear opinions on the relevance of pain/discomfort to T2D, while three did not clearly indicate whether the item was relevant. This item was considered relevant to T2D by nine participants. Three of these said the question was relevant to nerve pain or pain in their feet (e.g., *“I mean my own experience, discomfort when you maybe have this burning sensation in your feet”*), and another three said the question was relevant to pain/discomfort associated with medication (e.g., *“people who are on the injectable medications, they feel some pain and swelling as well”*; *“pain with the side effects of the medication”*). Three participants said this item was not relevant to T2D (e.g., “*with diabetes I don’t connect so much pain and discomfort; I connect more lack of energy*”). The remaining ten participants indicated that the item was relevant to other patients (*n* = 3; *“I connect pain and diabetes, but basically from what I've heard from other patients or about what other patients”*), severe patients (*n* = 5; e.g., *“I’ve had no pain or discomfort…I do know people who have suffered from pain and discomfort.”*), or for some patients (*n* = 2; *“It can be, but maybe not for most patients.”*).

#### Anxiety/depression

Twenty participants provided clear opinions on the relevance of anxiety/depression to T2D, while five did not clearly indicate whether the item was relevant. Anxiety/depression was the EQ-5D item that was considered relevant by the greatest number of participants (*n* = 17, 68 %; e.g., *“it’s something that’s never going to get better.... I find that makes me quite anxious about it.”*; *“It is your body and you’ve got a responsibility to make sure it’s running with a balance of medication, insulin if necessary, food, and energy. That responsibility can make you very anxious because it has to be the first and foremost of your day…It’s like carrying the world on your shoulder like Atlas sometimes”*). None of the participants said this item was irrelevant to T2D. Three participants (12.0 %) provided mixed responses indicating that the anxiety/depression item may be relevant for other T2D patients (*n* = 2; e.g., *“I’m not personally, but I know other people that do get depressed that they’re diabetics.”*) or that the item is relevant to general health but is not specific to T2D (*n* = 1; e.g., *“it's relevant to everybody and not just T2D.”*).

### Cognitive interviews: concepts not captured by the EQ-5D-5L

Most participants (92.0 %) reported that T2D and/or its treatment/monitoring requirements had an impact on their quality of life that was important to them but not captured by the EQ-5D-5L (see Table 4 for an extensive list of concepts and patients’ quotes). The most commonly reported missing concepts related to food awareness and restriction (*n* = 13). Eight respondents mentioned emotional functioning other than depression/anxiety. Eight participants discussed social/relationship functioning. Several participants (*n* = 11) suggested the questionnaire should ask about activities related to T2D at a greater level of detail than assessed by the Usual Activities item. Examples include exercise (*n* = 7), specific impact on work (*n* = 5), and specific leisure activities (*n* = 1).

**Table 4:** Concepts identified as missing or recommended for addition

Concepts	Participants reporting concept as missing n (%)	Examples of quotes from participants
**Activities**	**11 (44.0 %)**	
Exercise	7 (28.0 %)	• How much exercise you’re getting to get the sugar levels down…have you got your diabetes 2 under control with insulin, food, and exercise?
Leisure activities	1 (4.0 %)	• I know you have something about usual activities, but you know some people do other things like leisure activities, maybe the older folks might go to bingo. The younger folks might want to play football, and stuff like that. I know I see usual activities here, you should be a bit more specific in terms of sports.
Work	5 (20.0 %)	• How did it impact on your job, your relationship with your employer… you hear stories of people who get diagnosed and the employer suddenly finds a reason why they are no longer needed.
		• Does it affect your employment? Because if you take tablets then I know there’s certain jobs you can’t do.
**Comorbidities/Health problems**	**10 (40.0 %)**	
Amputations	1 (4.0 %)	• I’ve known [people] that have had some sort of medical amputations as a result of the diabetes.
Circulation/swelling	2 (8.0 %)	• A late friend of mine had multiple amputations because of the diabetes…And I think it’s probably because of the circulatory issues in relation to diabetes.
Cognitive impact	1 (4.0 %)	• I would put something there, how diabetes influences the mind and the workings of the mind…I used to have a good mind…but I know that I am more forgetful.
Headache	2 (8.0 %)	• Whether they have, um, severe migraines or headaches.
Heart problems	1 (4.0 %)	• Whether they have heart problems with their diabetes.
Tingling/pins	1 (4.0 %)	• Sensory feelings…when you feel a bit tingly in the hands.
Tiredness/energy	2 (8.0 %)	• It’s all to do with energy. Are you energetic? Are you very tired all the time?
Vision	7 (28.0 %)	• Vision is a paramount thing….And it’s not mentioned on here at all.
**Diabetes symptoms/Blood glucose**	**3 (12.0 %)**	
Frequent urination	1 (4.0 %)	• If you’re out and about you have to think of going and spending a penny all the time.
Hypoglycemia	2 (8.0 %)	• How do you feel when you have hypo…you can’t literally control nothing. If you have a bad one you lie on the floor. You could wet yourself, mess yourself.
Thirst	2 (8.0 %)	• My mouth gets really, really dry and my tongue…And you can’t do nothing.
**Diabetes treatment and monitoring**	**7 (28.0 %)**	
Medication use	4 (16.0 %)	• How easy do you find it to take your medication. Some people are fearful of injecting their medications and they just won’t take it. It could have had something about do you find your medications easy to take because some people don’t like swallowing pills.
Treatment satisfaction	2 (8.0 %)	• Are you happy with the treatment?
Side effects of T2D medication	1 (4.0 %)	• I worry about the side effects of the continuing [pharmaceutical treatment] on the body mass.
Blood glucose monitoring	1 (4.0 %)	• There’s an inconvenience in doing what you’re doing… When I’m trying to multi-task and do things and I’m thinking maybe I need to do that blood test.
**Emotional**	**8 (32.0 %)**	
Emotions (other than anxiety/depression)	7 (28.0 %)	• How being a diabetic has influenced your moods and your irritation? Are you more moody? Are you more irritable? Are you more on edge? Have you noticed any change in your reaction? Are you more impatient?
		• Your blood sugars can impact your moods. I’ve found that for me, that’s one of the signs that I need to think about my blood sugars and check that things are okay. And it doesn’t really bring that out in here as much, except for anxiety and depression. But sometimes moods don’t go as far as anxiety and depression.
Worry	3 (12.0 %)	• Diabetes can cause stress, stress and worry. And that has an impact on day to day life.
**Food awareness/Food restrictions**	**13 (52.0 %)**	
Dietary restriction	12 (48.0 %)	• Do you miss any food. Do you miss out on any food?…I mean you are restricted…it limits your choices…That doesn’t come under mobility or self-care, activities, walk.
		• I would have liked to see something on has your diet changed.
		• The impact of having to adjust your diet can be quite a large thing for many people.
		• I think you really should include diet.
Food awareness	5 (20.0 %)	• I started work at 5:00 this morning so I consciously eat something before I leave. Then I'm in meetings till 11:00, so I take something in my pocket to get me through the meetings…It comes back to the fact that it's constant vigilance.
**Infection**	**5 (20.0 %)**	
Infections	3 (12.0 %)	• If you cut yourself you're at an infection risk. And there should be something there in that.
Taking care of feet	2 (8.0 %)	• I feel I need to be very careful about my feet.
**Relationships and social life**	**8 (32.0 %)**	
Impacts on family	2 (8.0 %)	• I suppose you could ask how it affects your direct family or friends. Sometimes people can worry more about it because they don’t know what’s involved in it.
Relationships	3 (12.0 %)	• How it affects their relationships with their employers and relatives, acquaintances, whatever.
Sex life/sexual function	3 (12.0 %)	• It’s going to sound silly, sex life…Some people can get an erection and I can’t because of the diabetes.
Social	3 (12.0 %)	• If I do get problems with my blood sugar, it can cause me to be less sociable… and grumpy, which of course will impact on relationships with others.
		• The wider circle of people around you don’t understand it. And that can create pressures…and it leads to sort of antisocial behavior.
**Weight**	**4 (16.0 %)**	• You know because I don’t see nothing here about weight.
**Other responses**	**6 (24.0 %)**	
Financial	4 (16.0 %)	• It puts the cost up of insurance.
Healthcare	3 (12.0 %)	• How do you think your medical support has been since you’ve been diagnosed?… You could ask what support you’ve had from your practitioner.
Hereditary	2 (8.0 %)	• Possibly something around family history, because …it helps explain the sort of different reactions to it.
Positive impacts	2 (8.0 %)	• You could ask a general question, “Is there anything positive that’s come out of your diagnosis?” So if someone said they were grossly overweight and they’ve lost weight that’s positive isn’t it. If they’ve altered their diet to a healthier diet. If they’re doing more exercise. I mean they may have even taken up a different job or something because of the changes in their life, which is positive.

## Discussion

These qualitative findings add to published quantitative research to provide a more thorough understanding of the performance and relevance of the EQ-5D in patients with T2D. Results highlight the inherent strengths and limitations of a generic measure such as the EQ-5D. Generic preference-based instruments maximize applicability across populations and comparability across studies, but may be associated with limitations in specific patient groups. Given that the instrument was intended to provide a brief tool for quantifying health status across all populations, it is encouraging that about half of this T2D sample said the instrument was relevant to their own experience.

Findings also raise questions about the content validity of the EQ-5D-5L for this population. It is not surprising to find that this brief generic measure is missing content that could be important to a specific population. However, these qualitative results provide new insight into the specific ways that the EQ-5D may or may not be adequate for patients with T2D, while identifying specific missing concepts that are important to this patient group. Because the EQ-5D is so commonly used in this population, it is important to understand these potential limitations so that they may be addressed in future research and considered when selecting a utility assessment strategy.

When asked about the instrument’s relevance, a substantial portion of the sample provided mixed responses, stating that the EQ-5D could be relevant to other and possibly more severe patients, but not to their own personal experience. Furthermore, almost all respondents noted that important aspects of diabetes-specific quality of life were missing from the questionnaire. Current results add to a previous study in which generic preference-based measures including the EQ-5D were mapped onto a conceptual model of T2D, with results suggesting incomplete coverage of relevant concepts in disease-specific areas [21].

While the 2013 NICE guide suggests that content validity of the EQ-5D should be considered when deciding whether the instrument is appropriate for identifying utilities of a specific patient population, the guide does not discuss the level of content validity that should be considered acceptable. Therefore, it is difficult to know what standards the instrument should meet for this particular purpose. For example, in the current study, roughly half of the sample said the instrument was relevant to their own experience. Although this level of relevance would not be sufficient for a measure used to assess clinical trial outcomes, the purpose of a generic preference-based measure is different, and therefore, standards for content validity may also be different. A brief generic measure such as the EQ-5D is not designed to capture all relevant aspects of health for all patients. Instead, the instrument offers a classification system that may be used to characterize health across a broad range of the population so that response profiles can be valued and that utilities can be derived. While the instrument should capture the most important aspects of health, a brief generic instrument cannot be expected to capture every aspect of health status that has an impact on quality of life across all types of patients. When using a generic preference-based instrument to obtain utilities, researchers are likely aware that they are sacrificing some relevance and comprehensiveness in order to maximize comparability across studies.

In sum, the current findings highlight trade-offs researchers face when using a generic preference-based measure such as the EQ-5D. It would be useful to discuss these trade-offs in an attempt to reach consensus within the EuroQoL and health technology assessment (HTA) communities. Specifically, what degree of content validity and relevance is necessary for the EQ-5D to be considered appropriate for obtaining utilities within a specific patient population? Clearly, important aspects of almost any medical or psychiatric condition are likely to be missing from this brief instrument, and the adequacy of symptom and quality of life coverage will vary across patient populations. Symptoms and impact missing from the questionnaire may be important for one population, but irrelevant for another. Qualitative research, such as the current interviews, are useful for examining whether the EQ-5D is missing content that may be important to specific patient groups. After identifying this missing content, researchers can consider whether the excluded material significantly undermines the content validity of the instrument for the purpose of gathering utilities. If the missing content truly appears to be important, the qualitative findings can be used as justification for considering the EQ-5D to be “inappropriate” for the specific patient group and using another method to identify utilities or to measure the health status of a specific patient group.

When qualitative research suggests the EQ-5D may not have sufficient content validity in a specific patient population, a range of alternative approaches are available for utility assessment. If researchers want to minimize deviation from the EQ-5D, they may consider adding a condition-specific item to the five items of the EQ-5D [22–24]. This “bolt-on” approach has recently been used as a way to make the generic EQ-5D applicable to specific medical conditions not adequately covered in the standard five items. Another commonly used approach that maintains some comparability to the EQ-5D involves mapping (also called cross-walking) a condition-specific measure to the EQ-5D, although the adequacy of the mapping algorithm is limited in situations where the EQ-5D is not relevant for a specific patient population [25, 26]. For situations when the EQ-5D is unlikely to be relevant or sensitive, there are methods for identifying utilities associated with specific diseases or treatments, such as condition-specific preference-based measures [27] and studies in which respondents value health state descriptions (i.e., vignettes) designed to represent specific relevant characteristics. For example, the health state description approach has been used to estimate the disutility of treatment attributes specifically relevant to patients with T2D [28, 29]. While these methods are likely to be more sensitive to the utility impact of specific disease and treatment characteristics, their primary limitation in the HTA context is that comparability to utilities derived from generic preference-based instruments is uncertain.

Another approach that has been recommended is to administer the EQ-5D in conjunction with condition-specific instruments [17, 30, 31]. By administering both types of instruments, researchers can estimate utility with the EQ-5D while assessing specific symptoms and impact with the condition-specific measure. The correlation between the two types of measures may also provide information on the sensitivity and relevance of the EQ-5D within the target population.

Several limitations of the current study should be acknowledged. As a qualitative analysis with a small sample, the current study is particularly susceptible to selection biases, and results are not necessarily representative of the larger population of patients with T2D. Therefore, current findings are best suited for generating hypotheses and raising issues, and the current study should not be interpreted as a definitive assessment of the EQ-5D. In addition, the study was conducted with a convenience sample recruited via advertisements, rather than from clinics specializing in diabetes. It is not known whether this group of patients was systematically different from a sample that may be recruited in clinical settings. It is possible that the EQ-5D could be viewed as more relevant by a sample recruited in a clinic, which could have greater rates of severe diabetes than the current sample recruited via advertisements.

## Conclusions

Overall, results highlight benefits and limitations of generic preference-based measures such as the EQ-5D-5L. These instruments are short and easy to administer, and they efficiently provide an estimate of utility while maximizing comparability across utility scores that may be used in cost-utility models. On the other hand, these measures could be missing important condition-specific or treatment-specific content, and therefore, they may not be sufficiently relevant or sensitive for some patient populations. The current study identified specific content missing from the EQ-5D, which could limit content validity of the instrument when used in some patients with T2D. Therefore, it is recommended that researchers consider the advantages and disadvantages of various options when selecting utility assessment methods and when using utility values in models.

## References

[1] Clarke P, Gray A, Holman R (2002). Estimating utility values for health states of type 2 diabetic patients using the EQ-5D (UKPDS 62). Med Decis Making.

[2] Glasziou P, Alexander J, Beller E, Clarke P, Advance Collaborative Group (2007). Which health-related quality of life score? a comparison of alternative utility measures in patients with type 2 diabetes in the ADVANCE trial. Health Qual Life Outcomes.

[3] Grandy S, Fox KM, Shield Study Group (2012). Change in health status (EQ-5D) over 5 years among individuals with and without type 2 diabetes mellitus in the SHIELD longitudinal study. Health Qual Life Outcomes.

[4] Koopmanschap M (2002). Code- advisory board: coping with type II diabetes: the patient's perspective. Diabetologia.

[5] Reaney M, Mathieu C, Ostenson CG, Matthaei S, Krarup T, Kiljanski J, et al. Patient-reported outcomes among patients using exenatide twice daily or insulin in clinical practice in six European countries: the CHOICE prospective observational study. Health Qual Life Outcomes. 2013;11:217. 10.1186/1477-7525-11-217 PMC3900476 24369764

[6] U.K. Prospective Diabetes Study Group (1999). Quality of life in type 2 diabetic patients is affected by complications but not by intensive policies to improve blood glucose or blood pressure control (UKPDS 37). Diabetes Care.

[7] Janssen MF, Lubetkin EI, Sekhobo JP, Pickard AS (2011). The use of the EQ-5D preference-based health status measure in adults with Type 2 diabetes mellitus. Diabet Med.

[8] NICE (National Institute for Health and Care Excellence): Process and methods guides: Guide to the methods of technology appraisal 2013. London, UK: National Institute for Care Excellence (NICE); 2013. http://www.nice.org.uk/article/pmg9/chapter/Foreword. Accessed: August 19, 2013.

[9] Food & Drug Administration (2009). Guidance for Industry - Patient-Reported Outcome Measures: Use in Medical Product Development to Support Labeling Claims.

[10] Patrick DL, Burke LB, Gwaltney CJ, Leidy NK, Martin ML, Molsen E, et al. Content validity--establishing and reporting the evidence in newly developed patient-reported outcomes (PRO) instruments for medical product evaluation: ISPOR PRO Good Research Practices Task Force report: part 2--assessing respondent understanding. Value Health. 2011;14:978–88. 10.1016/j.jval.2011.06.013 22152166

[11] Patrick DL, Burke LB, Gwaltney CJ, Leidy NK, Martin ML, Molsen E, et al. Content validity--establishing and reporting the evidence in newly developed patient-reported outcomes (PRO) instruments for medical product evaluation: ISPOR PRO good research practices task force report: part 1--eliciting concepts for a new PRO instrument. Value Health. 2011;14:967–77. 10.1016/j.jval.2011.06.014 22152165

[12] Rothman M, Burke L, Erickson P, Leidy NK, Patrick DL, Petrie CD (2009). Use of existing patient-reported outcome (PRO) instruments and their modification: the ISPOR Good Research Practices for Evaluating and Documenting Content Validity for the Use of Existing Instruments and Their Modification PRO Task Force Report. Value Health.

[13] Brod M, Tesler LE, Christensen TL (2009). Qualitative research and content validity: developing best practices based on science and experience. Qual Life Res.

[14] Leidy NK, Vernon M. Perspectives on patient-reported outcomes: content validity and qualitative research in a changing clinical trial environment. Pharmacoeconomics. 2008;26:363–70. 10.2165/00019053-200826050-00002 18429654

[15] Janssen MF, Pickard AS, Golicki D, Gudex C, Niewada M, Scalone L, et al. Measurement properties of the EQ-5D-5L compared to the EQ-5D-3 L across eight patient groups: a multi-country study. Qual Life Res. 2013;22:1717–27. 10.1007/s11136-012-0322-4 PMC3764313 23184421

[16] Matza LS, Boye KS, Yurgin N, Brewster-Jordan J, Mannix S, Shorr JM, et al. Utilities and disutilities for type 2 diabetes treatment-related attributes. Qual Life Res. 2007;16:1251–65. 10.1007/s11136-007-9226-0 17638121

[17] Mulhern B, Meadows K (2014). The construct validity and responsiveness of the EQ-5D, SF-6D and Diabetes Health Profile-18 in type 2 diabetes. Health Qual Life Outcomes.

[18] Speight J, Reaney MD, Barnard KD (2009). Not all roads lead to Rome-a review of quality of life measurement in adults with diabetes. Diabet Med.

[19] Dolan P (1997). Modeling valuations for EuroQol health states. Med Care.

[20] Herdman M, Gudex C, Lloyd A, Janssen M, Kind P, Parkin D, et al. Development and preliminary testing of the new five-level version of EQ-5D (EQ-5D-5L). Qual Life Res. 2011;20:1727–36. 10.1007/s11136-011-9903-x PMC3220807 21479777

[21] McGrath C, Rofail D, Gargon E, Abetz L (2010). Using qualitative methods to inform the trade-off between content validity and consistency in utility assessment: the example of type 2 diabetes and Alzheimer's disease. Health Qual Life Outcomes.

[22] Longworth L, Yang Y, Young T, Mulhern B, Hernández Alava M, Mukuria C, et al. Use of generic and condition-specific measures of health-related quality of life in NICE decision-making: a systematic review, statistical modelling and survey. Health Technol Assess. 2014;18. 10.3310/hta18090 PMC4780954 24524660

[23] Swinburn P, Lloyd A, Boye KS, Edson-Heredia E, Bowman L, Janssen B (2013). Development of a disease-specific version of the EQ-5D-5L for Use in patients suffering from psoriasis: lessons learned from a feasibility study in the UK. Value in Health.

[24] Yang Y, Brazier J, Tsuchiya A (2014). Effect of adding a sleep dimension to the EQ-5D descriptive system: a "bolt-on" experiment. Med Decis Making.

[25] Brazier JE, Yang Y, Tsuchiya A, Rowen DL (2010). A review of studies mapping (or cross walking) non-preference based measures of health to generic preference-based measures. Eur J Health Econ.

[26] Longworth L, Rowen D (2013). Mapping to obtain EQ-5D utility values for use in NICE health technology assessments. Value Health.

[27] Brazier JE, Rowen D, Mavranezouli I, Tsuchiya A, Young T, Yang Y, et al. Developing and testing methods for deriving preference-based measures of health from condition-specific measures (and other patient-based measures of outcome). Health Technol Assess. 2012;16:1–114. 10.3310/hta16320 22832015

[28] Boye KS, Matza LS, Walter KN, Van Brunt K, Palsgrove AC, Tynan A (2011). Utilities and disutilities for attributes of injectable treatments for type 2 diabetes. Eur J Health Econ.

[29] Matza LS, Boye KS, Yurgin N (2007). Validation of two generic patient-reported outcome measures in patients with type 2 diabetes. Health Qual Life Outcomes.

[30] European Network for Health Technology Assessment (Eunethta). Endpoints used for relative effectiveness assessment of pharmaceuticals: Health-related quality of life and utility measures. February 2013; Final version.

[31] Fitzpatrick R, Davey C, Buxton MJ, Jones DR (1998). Evaluating patient-based outcome measures for use in clinical trials. Health Technol Assess.

